# Amiloidose Cardíaca e Inibidores de SGLT2: Sinais, Esperança e a Necessidade de Evidências Randomizadas

**DOI:** 10.36660/abc.20250894

**Published:** 2026-02-26

**Authors:** Humberto Villacorta, Aline Sterque Villacorta

**Affiliations:** 1 Universidade Federal Fluminense Niterói RJ Brasil Universidade Federal Fluminense, Niterói, RJ – Brasil

**Keywords:** Amiloidose, Inibidores do Transportador 2 de Sódio-Glicose, Insuficiência Cardíaca, Mortalidade

A insuficiência cardíaca secundária à amiloidose cardíaca continua sendo um dos cenários mais desafiadores na medicina cardiovascular contemporânea. Apesar dos grandes avanços nas terapias modificadoras da doença para amiloidose por transtirretina (ATTR) e por cadeias leves (AL), os desfechos ainda são amplamente determinados pela insuficiência cardíaca progressiva, disfunção renal e óbitos cardiovasculares.^1^ As terapias convencionais para insuficiência cardíaca são frequentemente mal toleradas ou ineficazes nessa cardiomiopatia restritiva, deixando os médicos com opções limitadas para melhorar o prognóstico. Nesse contexto, a análise de dados do mundo real realizada por Nunes et al. fornece evidências oportunas e instigantes que apoiam um papel potencial para os inibidores do cotransportador de sódio-glicose 2 (SGLT2) nessa população de alto risco.^2^

**Figura 1:**
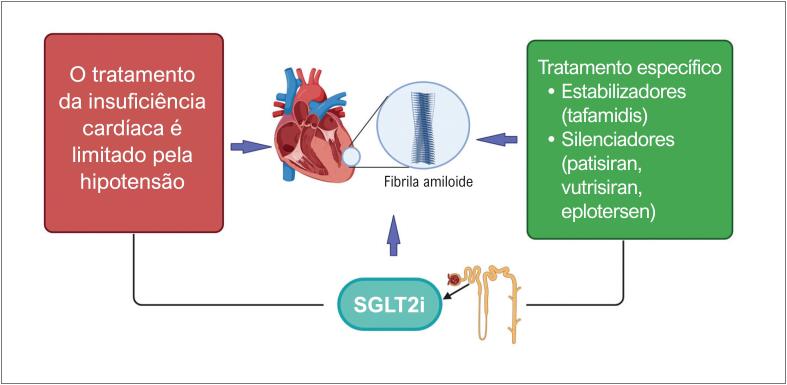
O possível papel dos inibidores de SGLT2 na amiloidose cardíaca. Pacientes com amiloidose geralmente são intolerantes ao tratamento clássico para insuficiência cardíaca. Como os inibidores de SGLT2 têm poucos efeitos hemodinâmicos, como hipotensão, representam uma boa opção para tratar pacientes com amiloidose que apresentam insuficiência cardíaca. Além disso, os inibidores de SGLT2 podem trazer benefícios na amiloidose cardíaca que vão além do tratamento da insuficiência cardíaca. IC: insuficiência cardíaca; SGLT2i: inibidores do cotransportador sódio-glicose 2.

Utilizando dados da rede colaborativa global TriNetX, os autores avaliaram mais de 2.700 pacientes com insuficiência cardíaca devido à amiloidose ATTR ou AL, pareados por escore de propensão. Em ambos os subtipos de amiloide, o tratamento com inibidores de SGLT2 foi consistentemente associado a reduções substanciais na mortalidade por todas as causas, hospitalização e eventos renais em 12 meses. A magnitude do benefício é impressionante: redução relativa de aproximadamente 50% na mortalidade e melhorias significativas tanto na insuficiência cardíaca quanto nos desfechos renais. Esses achados ampliam o crescente conjunto de dados observacionais que sugerem que os benefícios da inibição de SGLT2 podem transcender os fenótipos e etiologias tradicionais da insuficiência cardíaca e podem ter um papel específico na amiloidose cardíaca, como mostrado na Figura 1 .

Diversos aspectos deste estudo merecem destaque. Primeiramente, a inclusão tanto da amiloidose ATTR quanto da AL é extremamente relevante. Embora a cardiomiopatia ATTR tenha atraído maior atenção recentemente devido aos avanços no diagnóstico e tratamento, a amiloidose AL permanece particularmente letal, com desfechos intimamente ligados ao comprometimento cardíaco e renal. Demonstrar benefícios relativos semelhantes dos inibidores de SGLT2 em ambas as condições reforça a plausibilidade biológica de um efeito de classe que não depende da proteína precursora amiloide subjacente.

Em segundo lugar, o estudo aborda desfechos clinicamente relevantes. Mortalidade, hospitalização e eventos renais são precisamente os desfechos que mais importam para pacientes e médicos que tratam amiloidose. O efeito renoprotetor observado é especialmente notável, dada a alta prevalência de doença renal crônica tanto na amiloidose ATTR quanto na AL, e o papel central da disfunção renal na limitação das opções terapêuticas e na piora do prognóstico.

Do ponto de vista mecanístico, os benefícios observados são plausíveis. Os inibidores de SGLT2 exercem uma série de efeitos favoráveis, incluindo melhora da bioenergética miocárdica, redução do estresse oxidativo, modulação do manuseio intracelular de sódio e cálcio e potente ação renoprotetora.^3 , 4^ Na amiloidose – onde a disfunção diastólica, a sensibilidade ao volume e o comprometimento renal são características marcantes – esses mecanismos podem ser particularmente vantajosos. É importante ressaltar que os inibidores de SGLT2 são geralmente bem tolerados e hemodinamicamente neutros,^5 , 6^ características cruciais em pacientes que frequentemente não toleram vasodilatadores ou antagonistas neuro-hormonais.

Contudo, as limitações do estudo devem moderar nosso entusiasmo. Como se trata de uma análise retrospectiva baseada em registros eletrônicos de saúde, os resultados estão sujeitos a fatores de confusão residuais, apesar do rigoroso pareamento por escore de propensão. A falta de dados detalhados sobre a fração de ejeção do ventrículo esquerdo, o estágio da amiloidose, a classe funcional e a adesão ao tratamento limita uma fenotipagem mais aprofundada e insights mecanísticos. Além disso, a exposição ao tratamento foi inferida a partir das prescrições, e a interrupção durante o acompanhamento não pôde ser avaliada de forma confiável. Como em todos os estudos observacionais, a causalidade não pode ser estabelecida.

No entanto, é precisamente porque os pacientes com amiloidose cardíaca têm sido sistematicamente excluídos de ensaios clínicos randomizados sobre insuficiência cardíaca que evidências de alta qualidade do mundo real, como estas, se tornam tão valiosas. A consistência destes resultados com outros estudos observacionais e meta-análises recentes em cardiomiopatia ATTR^7 - 10^ reforça o sinal e sublinha uma necessidade urgente ainda não atendida: ensaios clínicos randomizados prospectivos com inibidores de SGLT2 especificamente concebidos para pacientes com amiloidose cardíaca.

Até que tais ensaios clínicos estejam disponíveis, os médicos precisam lidar com a incerteza. Os dados apresentados por Nunes et al. sugerem que, em pacientes cuidadosamente selecionados com insuficiência cardíaca devido à amiloidose ATTR ou AL, os inibidores de SGLT2 podem oferecer um benefício clínico significativo com um perfil de segurança aceitável. Embora ainda não sejam definitivos, esses achados apoiam o uso criterioso e individualizado de inibidores de SGLT2 como parte de uma estratégia abrangente para o tratamento da insuficiência cardíaca na amiloidose.

Em resumo, este estudo adiciona uma peça importante ao quebra-cabeça em evolução do manejo da amiloidose cardíaca. Ele desafia a antiga descrença terapêutica em torno do tratamento da insuficiência cardíaca nessa população e convida a comunidade cardiovascular a reconsiderar o papel dos inibidores de SGLT2 além das indicações convencionais. A mensagem é clara: a porta está aberta – agora é hora de os ensaios clínicos randomizados nos dizerem até onde podemos ir.
