# Supplementary material for: Amiloidose Cardíaca e Inibidores de SGLT2: Sinais, Esperança e a Necessidade de Evidências Randomizadas
Source: Arq Bras Cardiol. 2026 Feb 26;123(1):e20250894. [Article in Portuguese] doi: 10.36660/abc.20250894 (PMC13128188; doi:10.36660/abc.20250894)
Supplement: Translation

# Translation: Cardiac Amyloidosis and SGLT2 Inhibitors: Signal, Hope, and the Need for Randomized Evidence

**DOI:** 10.36660/abc.20250894i

**Authors:** Humberto Villacorta, Aline Sterque Villacorta

**Affiliations:** 1 Niterói RJ Brazil Universidade Federal Fluminense, Niterói, RJ – Brazil

**Keywords:** Amyloidosis, Sodium-Glucose Transporter 2 Inhibitors, Heart Failure, Mortality

Heart failure secondary to cardiac amyloidosis remains one of the most challenging scenarios in contemporary cardiovascular medicine. Despite major advances in disease-modifying therapies for both transthyretin (ATTR) and light-chain (AL) amyloidosis, outcomes are still largely driven by progressive heart failure, renal dysfunction, and cardiovascular deaths.^1^Conventional heart failure therapies are often poorly tolerated or ineffective in this restrictive cardiomyopathy, leaving clinicians with limited options to improve prognosis. In this context, the real-world analysis by Nunes et al. provides timely and provocative evidence supporting a potential role for sodium-glucose cotransporter 2 (SGLT2) inhibitors in this high-risk population.^2^

Using data from the TriNetX global collaborative network, the authors evaluated more than 2,700 propensity score-matched patients with heart failure due to ATTR or AL amyloidosis. Across both amyloid subtypes, treatment with SGLT2 inhibitors was consistently associated with substantial reductions in all-cause mortality, hospitalization, and renal events at 12 months. The magnitude of benefit is striking: approximately 50% relative reduction in mortality and meaningful improvements in both heart failure and renal outcomes. These findings extend the growing body of observational data suggesting that the benefits of SGLT2 inhibition may transcend traditional heart failure phenotypes and etiologies, and may have a specific role in cardiac amyloidosis, as shown in Figure 1 .

**Figure 1:** The possible role of SGLT2i in cardiac amyloidosis. Patients with amyloidosis are usually intolerant of the classic HF treatment. Since SGLT2i have little hemodynamic effects, such as hypotension, it is a good option to treat patients with amyloidosis who present with HF. Additionally, SGLT2i may have benefits in cardiac amyloidosis beyond the treatment of HF. HF: heart failure; SGLT2i: sodium-glucose cotransporter 2 inhibitors.

Several aspects of this study deserve emphasis. First, the inclusion of both ATTR and AL amyloidosis is highly relevant. While ATTR cardiomyopathy has recently attracted increased attention due to advances in diagnosis and treatment, AL amyloidosis remains particularly lethal, with outcomes closely linked to cardiac and renal involvement. Demonstrating similar relative benefits of SGLT2 inhibitors in both conditions strengthens the biological plausibility of a class effect that is not dependent on the underlying amyloid precursor protein.

Second, the study addresses clinically meaningful endpoints. Mortality, hospitalization, and renal events are precisely the outcomes that matter most to patients and clinicians managing amyloidosis. The observed renoprotective effect is especially noteworthy given the high prevalence of chronic kidney disease in both ATTR and AL amyloidosis and the central role of renal dysfunction in limiting therapeutic options and worsening prognosis.

Mechanistically, the benefits observed are plausible. SGLT2 inhibitors exert a constellation of favorable effects, including improved myocardial energetics, reduced oxidative stress, modulation of intracellular sodium and calcium handling, and potent renoprotective actions.^3 , 4^In amyloidosis – where diastolic dysfunction, volume sensitivity, and renal involvement are hallmarks – these mechanisms may be particularly advantageous. Importantly, SGLT2 inhibitors are generally well tolerated and hemodynamically neutral,^5 , 6^features that are critical in patients who often cannot tolerate vasodilators or neurohormonal antagonists.

Nonetheless, the study's limitations must temper our enthusiasm. As a retrospective analysis based on electronic health records, the findings are subject to residual confounding despite rigorous propensity score matching. The lack of detailed data on left ventricular ejection fraction, amyloid disease stage, functional class, and treatment adherence limits deeper phenotyping and mechanistic insights. Moreover, treatment exposure was inferred from prescriptions, and discontinuation during follow-up could not be reliably assessed. As with all observational studies, causality cannot be established.

Yet, it is precisely because patients with cardiac amyloidosis have been systematically excluded from randomized heart failure trials that high-quality real-world evidence such as this becomes so valuable. The consistency of these results with other recent observational studies and meta-analyses in ATTR cardiomyopathy^7 - 10^strengthens the signal and underscores an urgent unmet need: prospective randomized trials of SGLT2 inhibitors specifically designed for patients with cardiac amyloidosis.

Until such trials are available, clinicians are left to navigate uncertainty. The data presented by Nunes et al. suggest that, in carefully selected patients with heart failure due to ATTR or AL amyloidosis, SGLT2 inhibitors may offer meaningful clinical benefit with an acceptable safety profile. While not yet definitive, these findings support thoughtful, individualized use of SGLT2 inhibitors as part of a comprehensive heart failure strategy in amyloidosis.

In summary, this study adds an important piece to the evolving puzzle of cardiac amyloidosis management. It challenges the long-held therapeutic disbelief surrounding heart failure treatment in this population and invites the cardiovascular community to reconsider the role of SGLT2 inhibitors beyond conventional indications. The message is clear: the door is open – now it is time for randomized trials to tell us how far we can walk through it.
